# Vagus Nerve Stimulation (VNS) and Other Augmentation Strategies for Therapy-Resistant Depression (TRD): Review of the Evidence and Clinical Advice for Use

**DOI:** 10.3389/fnins.2018.00239

**Published:** 2018-04-10

**Authors:** Helge H. O. Müller, Sebastian Moeller, Caroline Lücke, Alexandra P. Lam, Niclas Braun, Alexandra Philipsen

**Affiliations:** ^1^Department of Psychiatry and Psychotherapy, Universitätsklinikum Bonn, Bonn, Germany; ^2^Department of Psychiatry and Psychotherapy, Carl von Ossietzky Universität Oldenburg, Oldenburg, Germany

**Keywords:** vagus nerve stimulation, therapy-resistant depression, neurostimulation, clinical practice, affective disorders

## Abstract

In addition to electroconvulsive therapy (ECT) and repetitive transcranial magnetic stimulation (rTMS), vagus nerve stimulation (VNS) is one of the approved neurostimulation tools for treatment of major depression. VNS is particularly used in therapy-resistant depression (TRD) and exhibits antidepressive and augmentative effects. In long-term treatment, up to two-thirds of patients respond. This mini-review provides a comprehensive overview of augmentation pharmacotherapy and neurostimulation-based treatment strategies, with a special focus on VNS in TRD, and provides practical clinical advice for how to select TRD patients for add-on neurostimulation treatment strategies.

## Introduction

Major depressive disease (MDD) is recognized worldwide as a frequently recurring or chronic and highly prevalent psychiatric disease (Beaucage et al., 2009; Maske et al., 2015). In addition to alterations in the typical domains of affective and mood symptoms, MDD is directly associated with high rates of suicidality and overall mortality as well as a well-established increased risk of death due to comorbid somatic disorders, such as myocardial infarction and stroke (Lasserre et al., 2017; Slepecky et al., 2017; Tesio et al., 2017; Vandeleur et al., 2017). Therefore, it has been projected that MDD will be the second leading cause of disability worldwide by the year 2020 (Michaud et al., 2001; Effinger and Stewart, 2012; Manetti et al., 2014). In addition to psychotherapeutic strategies, pharmacotherapy is usually used as a first-line treatment for MDD, yet many patients do not sufficiently respond to monotherapy with an established medication, such as a selective serotonin reuptake inhibitor (SSRI) (Fava and Davidson, 1996). Some progress has been made in developing safe and efficacious antidepressant treatments and novel pharmacotherapy-based treatment strategies, such as ketamine or selective NMDA receptor subtype 2B (NR2B) antagonists (Serafini et al., 2015; Andrade, 2017) with mechanisms other than monoamine neurotransmitter reuptake inhibition. Ketamine was found to quickly reduce depressive symptoms within hours of a single administration, thus further demonstrating the important role of glutamate in the development of depression (Serafini et al., 2014). However, data on the remission and recurrence rates of TRD under ketamine are still lacking. In summary, there currently seem to be no fundamental emerging innovations for the long-term treatment of MDD with antidepressant pharmacotherapy. Supportive, noninvasive add-on strategies, such as light-based therapy and exercise as well as alternative strategies, such as acupuncture and yoga, are used alongside pharmacological treatment strategies; however, their status within current treatment regimens is yet to be established, and many strategies are difficult to apply in an outpatient setting. Although evidence-based psychosocial interventions (Hunot et al., 2013; Hayes and Hofmann, 2017) are also under development, unfortunately, up to 50% of all patients with MDD do not achieve remission with currently available treatments (Zhou et al., 2015; Murphy et al., 2017). This subtype of MDD is classified as therapy-resistant depression (TRD) (Rush et al., 2006a,b; Mojtabai, 2017), which is defined by a lack of response or failure to fully respond or achieve remission after trials of at least two proven antidepressants with adequate dosing and duration (Bschor, 2010; Wiles et al., 2014; Holtzmann et al., 2016). At least one-third of all MDD patients are considered “therapy-resistant” (Rush et al., 2006a,b) (ongoing controversy discussed). Therefore, TRD disproportionally accounts for the largest proportion of the disease, underscoring the importance of innovative add-on therapy strategies for this particular type of TRD (McCullough, 2003; “Yoga for anxiety…”, 2009; Rizzo et al., 2011; Oldham and Ciraulo, 2014; Lucas et al., 2017; Sakurai et al., 2017).

Add-on or augmentation therapy means the combination of first-line antidepressive pharmacotherapy with a second treatment approach. In addition to pharmacological add-on therapy, neurostimulation techniques are increasingly used. Today, the most promising neurostimulation tools used to treat TRD are (1) Electroconvulsive therapy (ECT), (2) Transcranial direct current stimulation (tDCS), (3) Repetitive transcranial magnetic stimulation (rTMS), (4) Deep brain stimulation (DBS), (5) Magnetic seizure therapy (MST), (6) Cranial electrotherapy stimulation (CES), and (7) Vagus nerve stimulation (VNS). Each has a different application procedure, and there is a large variation in their effects and the clinical expertise required.

This mini-review provides a comprehensive overview of neurostimulation-based treatment strategies with a special focus on VNS in TRD and finally, aims to provide practical clinical advice for their use when selecting TRD patients for add-on neurostimulation treatment strategies.

## Adjunctive biological options for treating TRD alongside antidepressant pharmacotherapy

### Augmentation pharmacotherapy

#### Lithium

Lithium augmentation is (still) the state-of-the-art treatment in add-on and augmentative therapy with antidepressants when facing the challenge of TRD. Solid evidence from both large open-label and placebo-controlled trials highlights its efficacy in the treatment of resistant depression (Stage et al., 2007; Young, 2013; Nelson et al., 2014). Its notable effects include regulation of mood and circadian rhythms, and it also has a positive effect on suicidality and overall mortality. Lithium augmentation has significantly better antidepressant effects than the placebo, with a mean response rate of 41.2% (vs. 14.4%). Nevertheless, the risk of side effects (e.g., metabolic, cardiovascular, nephrologic) is significant, and its toxicity, especially when inadequate doses limit the clinical use of lithium, is notable (Edwards et al., 2013, 2014; Nelson et al., 2014; Hincapie-Castillo and Daniels, 2017).

#### Atypical antipsychotics

Atypical antipsychotics comprise the most-studied class of augmenting agents for SSRIs and serotonin-norepinephrine reuptake inhibitors (SNRIs) for depression (Kato and Chang, 2013; Fornaro et al., 2016; Bartoli et al., 2017). The FDA has approved both quetiapine and aripiprazole as well as the combination of olanzapine with fluoxetine for augmentation. Other agents include ziprasidone and risperidone, which have also been shown to be effective in treating MDD/TRD (Gabriel, 2013; Nelson, 2015).

Patients treated with atypical antipsychotics are approximately twice as likely to reach remission as patients treated with the placebo, as highlighted in several studies (De Fruyt et al., 2012; Spielmans et al., 2013; Wright et al., 2013; Fornaro et al., 2016). The use of atypical antipsychotics involves a careful risk-benefit assessment because these agents possess serious short- and long-term treatment-emergent (potentiated through combination therapies) side effects (e.g., sedation, central obesity, metabolic syndrome, and extrapyramidal side effects) (Shirzadi and Ghaemi, 2006; Fraguas et al., 2008; Temmingh, 2012; Sykes et al., 2017).

#### Thyroid augmentation

Thyroid hormones are an additional established option for the adjunctive treatment of TRD. Specifically, triiodothyronine (*T3*) is preferred for augmenting antidepressants due to its bioactivity in the CNS. In a meta-analysis of *T3* augmentation (25–50 μg/day) in probands who failed to respond to tricyclics, Aronson and colleagues found that *T3*-treated patients were twice as likely to respond as placebo-treated-patients (Aronson et al., 1996). In STAR^*^D, *T3* augmentation resulted in a 24.7% remission rate compared with a 15.9% remission rate for lithium augmentation in treatment-resistant patients who failed two previous antidepressant trials (Nierenberg et al., 2008; Warden et al., 2009). A disadvantage of T3 medication is its interference with thyroid metabolism in patients without hypothyroidism. Thus, treatment should be restricted to a few weeks, making this option unsuitable as a maintenance treatment (Cadieux, 1998).

#### Additional agents used for pharmacologic augmentation

A number of further drugs of diverse neuropsychopharmacological classes and properties are used as augmentation strategies of first-line antidepressive treatment for TRD. These drugs, which include bupropion, buspirone, methylphenidate, dopamine agonists, anticonvulsants, mirtazapine, modafinil, and pindolol (Dording, 2000), have been shown to possibly add to the antidepressive effect of first-line antidepressive treatment for TRD when administered in combination therapy. However, the scientific evidence for most of these agents is still comparably limited. In a recent meta-analysis of pharmacological augmentation strategies (Zhou et al., 2015), bupropion, buspirone, lamotrigine, methylphenidate, and pindolol all failed to show a superior effect compared to placebo.

### Neurostimulation options

Some promising neurostimulation tools for TRD in addition to VNS are described below.

ECT and rTMS (which has lower effect sizes) still stand as the gold standards for treatment with level I evidence (Pagnin et al., 2004; Minichino et al., 2012; Berlim et al., 2013b). MST and tDCS seem to be an option, especially when serious side effects occur during treatment with ECT. For DBS, the data are still limited due to small study groups, but the available data and experiences are promising.

### Electroconvulsive therapy (ECT)

ECT is the oldest neurostimulation therapy for treating TRD. It has been widely used in large-scale clinical studies of depression and has been found to be more effective than antidepressant drug use alone. It is also the most common therapeutic option for severe and recurrent depression when medication and psychotherapy have been unsuccessful (Kellner et al., 2012; Berlim et al., 2013b; Kellner, 2014). Based on solid data from six trials, a meta-analysis concluded that real ECT is significantly more effective than simulated (sham) ECT (standardized effect size 0.91, 95% CI −1.27 to −0.54) (The UK ECT Review Group, 2003).

Patients are given general anesthesia and a muscle relaxant before ECT and are continuously monitored throughout the procedure. Then, an electric current used to stimulate cerebral brain regions induces a generalized central seizure. The electrode placement is relevant to both efficacy and the development of side effects. The symmetric bitemporal electrode placement, which covers a large brain volume and induces a high level of seizure generalization, has high efficacy but produces more side effects than other placements. Unilateral ECT, in which the electrodes are placed on the right temple and to the right of the vertex, lowers the seizure generalization, efficacy and side effects (Calev et al., 1995; Prudic, 2008; Sidhom and Youssef, 2014; Muller et al., 2017b).

In clinical practice, the acute ECT treatment phase typically comprising 3 treatments/week can be followed by a taper phase with a reduction to 1–2x/week and then to 1x/week for several weeks. Many patients will then receive further maintenance ECT with a single treatment every 3–6 weeks. Importantly, there is no evidence for a need to limit the lifetime number of treatments in patients who need ongoing treatment (Kellner et al., 2012).

Overall, it can be concluded that ECT is a valid therapy for the treatment of TRD, including its severe and resistant forms. After remission, ECT is often replaced with maintenance ECT (mECT) to prevent relapse. However, good clinical outcomes, are diminished through high relapse rates of up to 50%” (Rifkin, 1988; Kho et al., 2003; Charlson et al., 2012; Pinna et al., 2016). Therefore, there is a 57% relapse rate with optimized pharmacotherapy and a 65% rate after a successful ECT series. The relapse rate remains 37% despite optimized pharmacotherapy and lavish and costly mECT sessions (Kellner et al., 2006; Eschweiler et al., 2007; Post et al., 2015).

### Magnetic seizure therapy (MST)

MST is a non-invasive convulsive neurostimulation therapy that induces an electric field in the brain and elicits a generalized tonic-clonic seizure. MST is being investigated as an alternative to ECT for use under general anesthesia with assisted ventilation and continuous electroencephalographic (EEG) monitoring. MST has the potential for fewer side effects, such as cognitive dysfunction, than ECT (Lisanby et al., 2003; Allan and Ebmeier, 2011), but optimal stimulation parameters for MST are still being investigated. Most studies have used a coil placed at the vertex with a frequency of stimulation of 100 Hz, a pulse width of 0.2–0.4 ms, and a stimulation duration of 10 s (Kito, 2017). There are no large-scale studies comparing MST to sham stimulation and no large-scale controlled studies of relapse following maintenance MST (mMST) with regard to prevention strategies, so the therapy is still in the experimental stage (Allan and Ebmeier, 2011).

### Transcranial direct current stimulation (tDCS)

In tDCS, cortical areas are stimulated non-invasively via a low-intensity direct current. Stimulation via sponge-based rectangular pads lasts for 10–20 min and modulates the neuronal excitability in target cerebral regions (Tschirdewahn et al., 2015; Palm et al., 2016b). The stimulation is focused on the left dorsolateral prefrontal cortex region (DLPFC) to minimize hypo-activity of the left DLPFC, which is a main target region in depression (Berlim et al., 2013a; Dell'Osso and Altamura, 2014; Meron et al., 2015). This therapy has almost no side effects and is well tolerated among all treatment groups. Stimulation of cortical regions may result in changes in membrane resting potentials and modify synaptic transmission in the DLPFC, which ultimately results in a significant, but only moderate, reduction of depression (Liebetanz et al., 2006; Palm et al., 2016a).

### Repetitive transcranial magnetic stimulation (rTMS)

Clinically used since the mid-80s, rTMS delivers external magnetic pulses to the cortex. These pulses induce an electrical potential in the brain tissue that depolarizes target neurons (Bulteau et al., 2017; McClintock et al., 2018). Stimulation can be high frequency (1 Hz) or low frequency (<1 Hz), and rTMS can also be used in the form of maintenance rTMS (mrTMS) (Rachid, 2018). Low-frequency rTMS inhibits certain cortical regions, whereas high-frequency rTMS activates the stimulated regions (Baeken et al., 2009; Bakker et al., 2015). It has been used to reduce depression, even in patients with medication-resistant major depression, with very few side effects and up to a 60% response rate, but has only a small antidepressant effect during follow-up after short and acute treatment in the absence of active maintenance treatment (Dell'osso et al., 2011; Kedzior et al., 2015). Similarly, rTMS response rates are poor in patients for whom ECT has failed (Kedzior et al., 2017). These findings indicate that rTMS should be considered prior to pursuing ECT or as an add-on strategy and that patients who have not responded to ECT are unlikely to respond to rTMS treatment sessions alone (McClintock et al., 2018). The side effects of rTMS are mild and of short duration. Therefore, rTMS is a therapy that can be used for common depression treatment and is beneficial when combined with other standard treatments, such as pharmacotherapy and/or psychotherapy and other neurostimulation options (Perera et al., 2016). In recent years, there has also been growing evidence that, in addition to improvement of mood, rTMS might have a positive effect on cognitive functioning, which is often significantly reduced in patients with major depression. Aspects of cognitive performance reported to improve under rTMS include verbal memory, executive functioning, visuospatial ability, and recognition of facial expressions (Demirtas-Tatlidede et al., 2013). This may be an important advantage of rTMS, since cognitive impairment in MDD is insufficiently targeted by many other treatment options.

### Deep brain stimulation (DBS)

DBS is an invasive neurosurgical procedure for TRD. The targeted approach involves stereotaxic placement of unilateral and/or bilateral electrodes in predefined brain regions. These electrodes are then connected to an implanted neurostimulator. Although the mode of action remains unclear, it is hypothesized that chronic, high-frequency stimulation (130–185 Hz) reduces cerebral neural transmission by inactivating voltage-dependent ion channels and clinically restores the activity of specific neuronal circuits involved in TRD (“Deep brain stimulation…”, 2010; Cusin and Dougherty, 2012; Berlim et al., 2014). The targeted regions include the inferior thalamic peduncle, nucleus accumbens, lateral habenula, ventral striatum and subgenual cingulate cortex. Depending on the regions of interest, DBS is supposed to have antidepressant, strong antianhedonic, and antianxiety effects in TRD patients. It results in improvements related to social functioning, physical health and mood and anhedonic symptoms within TRD (Buhmann et al., 2017). No significant adverse effects of DBS (when implanted) have been recorded, thus highlighting DBS as promising in serious and chronic TRD. However, at this time only few clinical data sets with small sample sizes are available because the procedure is complex and requires direct brain surgery (Schlaepfer and Lieb, 2005; Kennedy et al., 2011; Jiménez et al., 2013; Lozano and Lipsman, 2013).

### Cranial electrotherapy stimulation (CES)

In pulsed CES, low-amplitude electric currents (<1 mA) are broadly applied to the brain via scalp electrodes. CES has been approved for the treatment of anxiety, depression, and insomnia by the FDA (Gilula and Barach, 2004; Gunther and Phillips, 2010; Kavirajan et al., 2014). CES may affect the reticular activating system, the limbic system, and the hypothalamus (Kirsch and Nichols, 2013). How CES exerts its antidepressant effect is not fully understood. A recent study showed that CES could deactivate cortical brain activity and alter connectivity in the default-mode network (Kavirajan et al., 2014). Clinically, CES also seems to decrease comorbid depression in anxiety disorders (Feusner et al., 2012; Kirsch et al., 2014). However, a Cochrane library review indicates that methodologically rigorous studies of the antidepressant effects of CES in the treatment of acute depression are still lacking (Kavirajan et al., 2014). How CES modulates underlying neuroplasticity or signaling pathways also needs clarification.

### Vagus nerve stimulation (VNS)

After decades of animal experimentation and application and after significant reductions in the frequency and severity of seizures were observed in response to stimulation of the vagus nerve, VNS was first applied in a human case of refractory epilepsy in 1988 (Rutecki, 1990; Uthman et al., 1990). VNS was then commercially approved for treatment of resistant epilepsy in 1997 (McLachlan, 1997; DeGiorgio et al., 2000; Henry, 2002). After showing its remarkable antidepressive clinical mode of action in a spin-off study and other controlled studies of TRD, it received approval for TRD in Europe and Canada in 2001–2005 (Sackeim et al., 2001; Topfer and Hailey, 2001; Marangell et al., 2002; Kosel and Schlaepfer, 2003). The therapy was then approved by the FDA for chronic depression and TRD in patients aged 18 years or older who do not respond to other antidepressant treatments (Nahas et al., 2006). Over 100,000 patients/year (both neurological and psychiatric indications) are treated worldwide (Cusin and Dougherty, 2012).

Surgical implantation is achieved by means of minor surgery, mainly neurosurgical, or otolaryngologic (Ng et al., 2010; Elliott et al., 2011). VNS requires an implantable pulse generator, which is surgically inserted under the skin of the chest and connected to an electrode placed in one of the vagus fibers in the neck. The repeatedly stimulated vagus nerve sends impulses from the periphery, where the electrode is placed, to the brain. Electrical stimulation of the vagus nerve centrally stimulates the nucleus tractus solitarius, which in turn is able to modulate multiple regions of the brain via its neuronal connections to anatomically distributed cortical and subcortical regions of the brain, the raphe nuclei and locus coeruleus, especially the limbic system. The right vagus nerve is not used because of the risk of potential severe bradycardia or arrhythmias. The left vagus nerve, whose fibers point to the central region, is used in VNS, which mainly stimulates the afferent fibers that communicate with the target regions to achieve improvement in mood. Therefore, this location is responsible for one of the main clinical effects of VNS.

In its mode of action, VNS modulates the concentrations of neurotransmitters (especially serotonin, norepinephrine, GABA and glutamate) and their metabolites while producing changes in the functional activity of CNS regions, which makes the mode of action of VNS similar to that of most antidepressants. Neuroimaging studies have shown evidence that activity in the thalamus and cortex in depressed patients is altered by VNS therapy. Changed activity in the orbital and ventromedial prefrontal cortices has also been recorded (Chae et al., 2003; Muller et al., 2013b). The most frequent acute complications of VNS implantation include temporary salivation, coughing, paralysis of the vocal cords, lower facial weakness, rarely bradycardia, and, very rarely, asystole; all side effects are generally fully reversible (Elliott et al., 2011; Schneider et al., 2015).

In a nutshell, there is growing and promising evidence for the use of VNS for depression in a 12-month trial. In a recent double-blind trial with 331 TRD patients, adjunct VNS at low (0.25 mA, 130 ls pulse width), medium (0.5–1.0 mA, 250 ls), and high (1.25–1.5 mA, 250 ls) currents was effective over 1 year (Aaronson et al., 2013; Feldman et al., 2013; Muller et al., 2013a). Smaller studies also showed high levels of remittance of TRD over longer periods (>5 y) (Muller et al., 2013a, 2017a). Recently, Aaronson et al. provided a large set of data showing improved outcomes for adjunctive VNS observed in both ECT responders and non-responders. Within the D-23 VNS registry (489 in the VNS arm and 276 in the treatment-as-usual arm), cumulative remission, based on an MADRS total score, demonstrated that over time, patients in the VNS arm were significantly more likely to experience remission than those in the treatment-as-usual arm (43.3 and 25.7%, respectively), demonstrating significant efficacy. The MADRS is a popular scale because of its high inter-rater reliability and high sensitivity to detect changes in treatment effects. Due to these features, the MADRS has been widely used in mood disorder studies. Higher scores indicate greater symptom severity. As demonstrated in previous studies, the scale has good parallel form reliability. The 5-year cumulative response rate for patients in the VNS arm who had previously responded to ECT was 71.3% compared with 56.9% for the ECT responders in the treatment-as usual arm. For ECT non-responders in the VNS arm, the response rate was 59.6%, compared with 34.1% (95% for ECT non-responders in the treatment-as usual arm). These results show that VNS is promising, particularly, but not only, as a feasible adjunctive tool for ECT responders (Aaronson et al., 2017). In addition to the antidepressive mode of action, a remarkable finding is that VNS seems to have a specific lower all-cause mortality rate and an anti-suicidal effect (Aaronson et al., 2013, 2017; Berry et al., 2013). Therefore, the longer-term results of VNS are encouraging, and VNS can be considered for patients with chronic depression, particularly in situations where treatment resistance may be an issue. A limitation of the available studies on VNS stimulation cited above is the lack of a control group receiving sham stimulation. Sham stimulation is used as a placebo treatment in neurostimulation trials, i.e., specific sham coils, which mimic the feeling of the real stimulation procedure, are used in randomized controlled rTMS trials. Sham stimulation in VNS treatment is much more problematic on an ethical level not only because surgery is required but also because a long period of >6 months of sham stimulation would be required due to the delayed entry of treatment effects under VNS. This seems unethical in light of the seriousness of MDD, including the possible risk of suicide (Aaronson et al., 2013). Thus, the possibility cannot be excluded that a placebo effect influenced the results of the studies cited above. Nonetheless, due to the solid magnitude of effects and the addition of a control group receiving other antidepressive treatment to the large D-23 registry trial (Aaronson et al., 2017), it seems unlikely that the observed effects were due to the placebo effect alone.

## Conclusion

### Selection of patients for adjunctive neurostimulation

**Figure 1:**
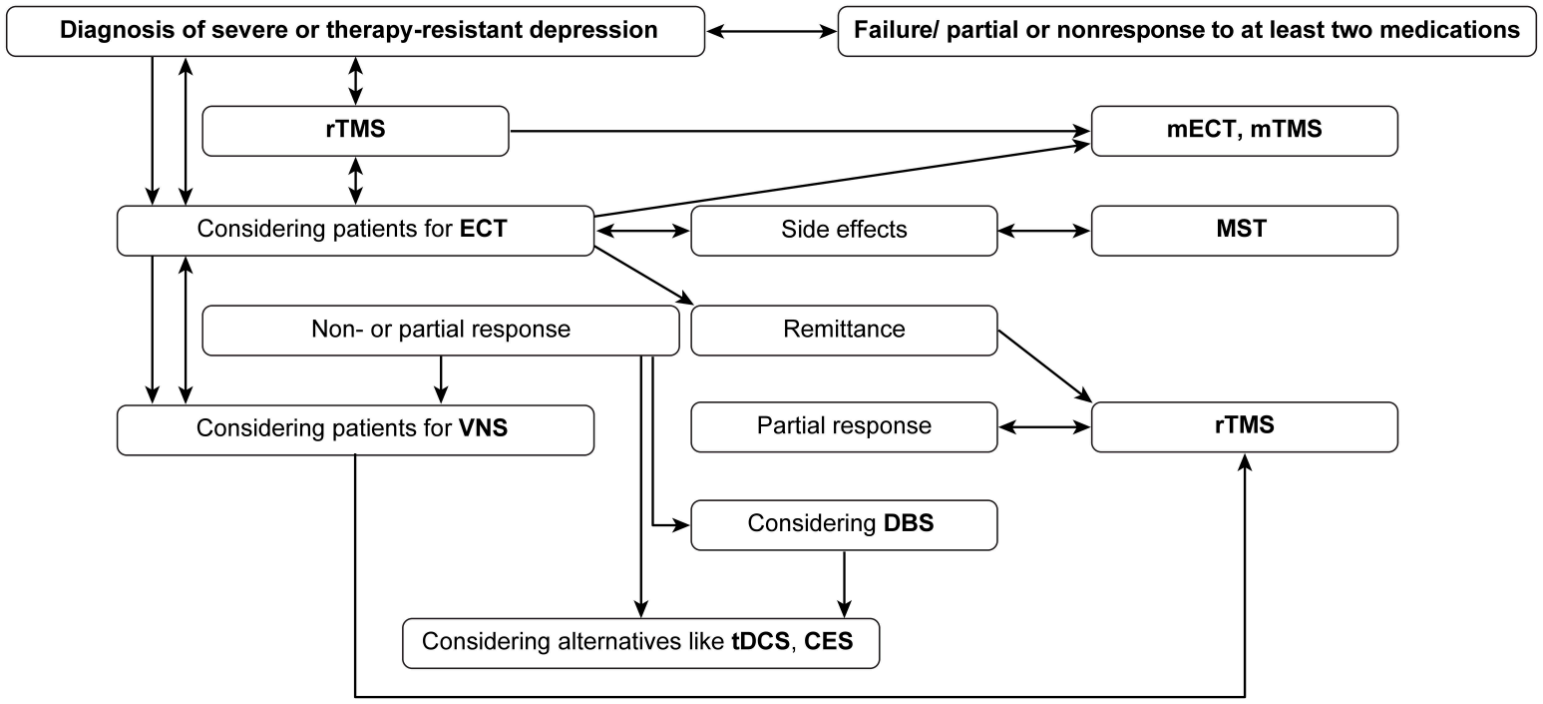
Clinical pathways when choosing neurostimulation techniques.

The harm of chronic and TRD highlights the need for evidence-based adjunctive treatment options. ECT and others, especially/in addition to rTMS, are primarily delivered for seriously ill depressed probands. Alternative and/or add-on strategies, such as DBS or VNS, should be strongly recommended to patients (Table 1, Figure 1) as promising adjunctive options to ECT (the gold standard), especially when treatment resistance occurs. Additionally, the combination of rTMS and ECT is promising, and when side effects of ECT occur, MST is a possible alternative. Only ECT and rTMS have level I evidence for regular treatment; VNS is also approved for the indication group for which r-TMS and CES are FDA-approved.

**Table 1:** Neurostimulation options for treatment of TRD.

**Technique**	**Main stimulation target region**	**Mode of action**	**Evidence**	**Pro**	**Con**
ECT	Cerebral cortex	Small currents and generalized seizure induction	Strong	First line therapy for patients who failed in pharmacotherapy, rapid antidepressive effects, long-lasting clinical experiences	Relapse rates, effort, cognitive side effects
tDCS	Cerebral cortex	Anode and cathode sending constant low current (0.5–2 mA) directly to the brain	Weak-moderate	Non-invasive, rapid effects	Less clinical experience
rTMS	Cerebral cortex	Magnetic pulses to depolarize cerebral neurons	Strong	Non-invasive, approved	Relapse rates, effort, small effect sizes
DBS	Nucleus accumbens, lateral habenula, ventral striatum, inferior thalamic nucleus, peduncle, subgenual cingulate	High-frequency stimulation (130–185 Hz); reduction of neuronal transmission by inactivating voltage-dependent ion channels; modulation of neuronal circuits	Moderate, experimental	Probably highly effective	Implantation procedure
MST	Cerebral cortex	Based on ECT, probably effects increased glucose metabolism	Weak-moderate	Less side effects than ECT	No broad evidence
CES	Probably affects limbic system, reticular activating system, hypothalamus	Electrical currents (<1 mA)	Weak-moderate	Non-invasive, supposed antidepressive mode of action, FDA-approved	No broad evidence
VNS	Left peripheral vagus nerve	(Long-term) modulation of neurotransmitters	Moderate-strong	Anti-suicidal effects and rates of remittance, combination option with nearly all other treatment options, FDA-approved	Latency in antidepressive efficacy

Compared to other neurostimulation techniques, VNS has the advantages of more solid scientific evidence for efficacy compared to MST, tDCS and CES and, after initial implantation, a comparably small burden of time and effort for maintenance treatment compared to ECT and rTMS. Compared to maintenance ECT, VNS is also less invasive in the long term. However, a disadvantage of VNS is the delay of effects after implantation, with substantial treatment effects often only occurring after 3–12 months of treatment.

For MST, tDCS, and CES as adjunctive treatments alone, there is not yet sufficient evidence to recommend them in the first line, but as add-on strategies, they probably should be considered.

In summary, it seems that a special future focus should be placed on therapy based on powerful (especially when combined) augmentative neurostimulation options. Particularly because of the promising results from neurostimulation combination strategies (e.g., ECT followed by VNS and ECT/r-TMS), the expected augmentation effects of combining neurostimulation techniques should be strictly further evaluated in future controlled clinical studies.

## Author contributions

HM and AP: Conceived the review's focus; HM, SM, AL, CL, and NB: Conducted the literature review; SM, HM, and NB: Designed the tables and figures; HM and AP: Wrote the first draft, summarized, and finalized the manuscript. All the authors critically commented on drafts, gave expert opinions on neurostimulation and approved the final manuscript.

### Conflict of interest statement

HM received travel grants and speaker's compensation from LivaNova Germany within the last year. The other authors declare that the research was conducted in the absence of any commercial or financial relationships that could be construed as a potential conflict of interest.

## References

[B1] (2009). Yoga for anxiety and depression. Studies suggest that this practice modulates the stress response. Harv. Ment. Health Lett. 25, 4–5. 19475766

[B2] (2010). Deep brain stimulation shows promise for Alzheimer's, depression treatment. Doctors also now have more options in how DBS is used to treat Parkinson's disease. Duke Med. Health News 16, 1–2. 21186495

[B3] Aaronson S. T. Carpenter L. L. Conway C. R. Reimherr F. W. Lisanby S. H. Schwartz T. L.. (2013). Vagus nerve stimulation therapy randomized to different amounts of electrical charge for treatment-resistant depression: acute and chronic effects. Brain Stimul. 6, 631–640. 10.1016/j.brs.2012.09.013 23122916

[B4] Aaronson S. T. Sears P. Ruvuna F. Bunker M. Conway C. R. Dougherty D. D.. (2017). A 5-year observational study of patients with treatment-resistant depression treated with vagus nerve stimulation or treatment as usual: comparison of response, remission, and suicidality. Am. J. Psychiatry 174, 640–648. 10.1176/appi.ajp.2017.16010034 28359201

[B5] Allan C. L. Ebmeier K. P. (2011). The use of ECT and MST in treating depression. Int. Rev. Psychiatry 23, 400–412. 10.3109/09540261.2011.614223 22200130

[B6] Andrade C. (2017). Ketamine for depression, 4: in what dose, at what rate, by what route, for how long, and at what frequency? J. Clin. Psychiatry 78, e852–e857. 10.4088/JCP.17f11738 28749092

[B7] Aronson R. Offman H. J. Joffe R. T. Naylor C. D. (1996). Triiodothyronine augmentation in the treatment of refractory depression. A meta-analysis. Arch. Gen. Psychiatry 53, 842–848. 10.1001/archpsyc.1996.01830090090013 8792761

[B8] Baeken C. De Raedt R. Van Hove C. Clerinx P. De Mey J. Bossuyt A. (2009). HF-rTMS treatment in medication-resistant melancholic depression: results from 18FDG-PET brain imaging. CNS Spectr. 14, 439–448. 10.1017/S1092852900020411 19890238

[B9] Bakker N. Shahab S. Giacobbe P. Blumberger D. M. Daskalakis Z. J. Kennedy S. H. (2015). rTMS of the dorsomedial prefrontal cortex for major depression: safety, tolerability, effectiveness, and outcome predictors for 10 Hz vs. intermittent theta-burst stimulation. Brain Stimul. 8, 208–215. 10.1016/j.brs.2014.11.002 25465290

[B10] Bartoli F. Dell'Osso B. Crocamo C. Fiorillo A. Ketter T. A. Suppes T.. (2017). Benefits and harms of low and high second-generation antipsychotics doses for bipolar depression: a meta-analysis. J. Psychiatr. Res. 88, 38–46. 10.1016/j.jpsychires.2016.12.021 28086127

[B11] Beaucage C. Cardinal L. Kavanagh M. Aubé D. (2009). Major depression in primary care and clinical impacts of treatment strategies: a literature review. Sante. Ment. Que. 34, 77–100. 10.7202/029760ar 19475195

[B12] Berlim M. T. McGirr A. Van den Eynde F. Fleck M. P. Giacobbe P. (2014). Effectiveness and acceptability of deep brain stimulation (DBS) of the subgenual cingulate cortex for treatment-resistant depression: a systematic review and exploratory meta-analysis. J. Affect. Disord. 159, 31–38. 10.1016/j.jad.2014.02.016 24679386

[B13] Berlim M. T. Van den Eynde F. Daskalakis Z. J. (2013a). Clinical utility of transcranial direct current stimulation (tDCS) for treating major depression: a systematic review and meta-analysis of randomized, double-blind and sham-controlled trials. J. Psychiatr. Res. 47, 1–7. 10.1016/j.jpsychires.2012.09.025 23084964

[B14] Berlim M. T. Van den Eynde F. Daskalakis Z. J. (2013b). Efficacy and acceptability of high frequency repetitive transcranial magnetic stimulation (rTMS) versus electroconvulsive therapy (ECT) for major depression: a systematic review and meta-analysis of randomized trials. Depress. Anxiety 30, 614–623. 10.1002/da.22060 23349112

[B15] Berry S. M. Broglio K. Bunker M. Jayewardene A. Olin B. Rush A. J. (2013). A patient-level meta-analysis of studies evaluating vagus nerve stimulation therapy for treatment-resistant depression. Med. Devices 6, 17–35. 10.2147/MDER.S41017 23482508 PMC3590011

[B16] Bschor T. (2010). Therapy-resistant depression. Expert Rev. Neurother. 10, 77–86. 10.1586/ern.09.137 20021322

[B17] Buhmann C. Huckhagel T. Engel K. Gulberti A. Hidding U. Poetter-Nerger M.. (2017). Adverse events in deep brain stimulation: a retrospective long-term analysis of neurological, psychiatric and other occurrences. PLoS ONE 12:e0178984. 10.1371/journal.pone.0178984 28678830 PMC5497949

[B18] Bulteau S. Sébille V. Fayet G. Thomas-Ollivier V. Deschamps T. Bonnin-Rivalland A.. (2017). Efficacy of intermittent Theta Burst Stimulation (iTBS) and 10-Hz high-frequency repetitive transcranial magnetic stimulation (rTMS) in treatment-resistant unipolar depression: study protocol for a randomised controlled trial. Trials 18:17. 10.1186/s13063-016-1764-8 28086851 PMC5237321

[B19] Cadieux R. J. (1998). Practical management of treatment-resistant depression. Am. Fam. Physician 58, 2059–2062. 9861879

[B20] Calev A. Gaudino E. A. Squires N. K. Zervas I. M. Fink M. (1995). ECT and non-memory cognition: a review. Br. J. Clin. Psychol. 34 (Pt 4), 505–515. 10.1111/j.2044-8260.1995.tb01485.x 8563658

[B21] Chae J. H. Nahas Z. Lomarev M. Denslow S. Lorberbaum J. P. Bohning D. E.. (2003). A review of functional neuroimaging studies of vagus nerve stimulation (VNS). J. Psychiatr. Res. 37, 443–455. 10.1016/S0022-3956(03)00074-8 14563375

[B22] Charlson F. Siskind D. Doi S. A. McCallum E. Broome A. Lie D. C. (2012). ECT efficacy and treatment course: a systematic review and meta-analysis of twice vs. thrice weekly schedules. J. Affect. Disord. 138, 1–8. 10.1016/j.jad.2011.03.039 21501875

[B23] Cusin C. Dougherty D. D. (2012). Somatic therapies for treatment-resistant depression: ECT, TMS, VNS, DBS. Biol. Mood Anxiety Disord. 2:14. 10.1186/2045-5380-2-14 22901565 PMC3514332

[B24] De Fruyt J. Deschepper E. Audenaert K. Constant E. Floris M. Pitchot W.. (2012). Second generation antipsychotics in the treatment of bipolar depression: a systematic review and meta-analysis. J. Psychopharmacol. 26, 603–617. 10.1177/0269881111408461 21940761

[B25] DeGiorgio C. M. Schachter S. C. Handforth A. Salinsky M. Thompson J. Uthman B.. (2000). Prospective long-term study of vagus nerve stimulation for the treatment of refractory seizures. Epilepsia 41, 1195–1200. 10.1111/j.1528-1157.2000.tb00325.x 10999559

[B26] Dell'Osso B. Altamura A. C. (2014). Transcranial brain stimulation techniques for major depression: should we extend TMS lessons to tDCS? Clin. Pract. Epidemiol. Ment. Health 10, 92–93. 10.2174/1745017901410010092 25317200 PMC4192830

[B27] Dell'osso B. Camuri G. Castellano F. Vecchi V. Benedetti M. Bortolussi S.. (2011). Meta-review of metanalytic studies with repetitive transcranial magnetic stimulation (rTMS) for the treatment of major depression. Clin. Pract. Epidemiol. Ment. Health 7, 167–177. 10.2174/1745017901107010167 22135698 PMC3227860

[B28] Demirtas-Tatlidede A. Vahabzadeh-Hagh A. M. Pascual-Leone A. (2013). Can noninvasive brain stimulation enhance cognition in neuropsychiatric disorders? Neuropharmacology 64, 566–578. 10.1016/j.neuropharm.2012.06.020 22749945 PMC3725288

[B29] Dording C. M. (2000). Antidepressant augmentation and combinations. Psychiatr. Clin. North Am. 23, 743–755. 10.1016/S0193-953X(05)70195-7 11147245

[B30] Edwards S. J. Hamilton V. Nherera L. Trevor N. (2013). Lithium or an atypical antipsychotic drug in the management of treatment-resistant depression: a systematic review and economic evaluation. Health Technol. Assess. 17, 1–190. 10.3310/hta17540 PMC4781298 24284258

[B31] Edwards S. J. Wakefield V. Nherera L. Trevor N. (2014). Systematic review and mixed treatment comparison of lithium or an atypical anti-psychotic (AAP) used to augment a selective serotonin reuptake inhibitor (SSRI) in treatment resistant depression (TRD). Value Health 17:A455. 10.1016/j.jval.2014.08.1242 27201261

[B32] Effinger J. M. Stewart D. G. (2012). Classification of co-occurring depression and substance abuse symptoms predicts suicide attempts in adolescents. Suicide Life Threat. Behav. 42, 353–358. 10.1111/j.1943-278X.2012.00092.x 22533529

[B33] Elliott R. E. Morsi A. Tanweer O. Grobelny B. Geller E. Carlson C.. (2011). Efficacy of vagus nerve stimulation over time: review of 65 consecutive patients with treatment-resistant epilepsy treated with VNS > 10 years. Epilepsy Behav. 20, 478–483. 21296622 10.1016/j.yebeh.2010.12.042

[B34] Eschweiler G. W. Vonthein R. Bode R. Huell M. Conca A. Peters O.. (2007). Clinical efficacy and cognitive side effects of bifrontal versus right unilateral electroconvulsive therapy (ECT): a short-term randomised controlled trial in pharmaco-resistant major depression. J. Affect. Disord. 101, 149–157. 10.1016/j.jad.2006.11.012 17196664

[B35] Fava M. Davidson K. G. (1996). Definition and epidemiology of treatment-resistant depression. Psychiatr. Clin. North Am. 19, 179–200. 10.1016/S0193-953X(05)70283-5 8827185

[B36] Feldman R. L. Dunner D. L. Muller J. S. Stone D. A. (2013). Medicare patient experience with vagus nerve stimulation for treatment-resistant depression. J. Med. Econ. 16, 62–74. 10.3111/13696998.2012.724745 22954061

[B37] Feusner J. D. Madsen S. Moody T. D. Bohon C. Hembacher E. Bookheimer S. Y.. (2012). Effects of cranial electrotherapy stimulation on resting state brain activity. Brain Behav. 2, 211–220. 10.1002/brb3.45 22741094 PMC3381625

[B38] Fornaro M. Stubbs B. De Berardis D. Perna G. Valchera A. Veronese N.. (2016). Atypical antipsychotics in the treatment of acute bipolar depression with mixed features: a systematic review and exploratory meta-analysis of placebo-controlled clinical trials. Int. J. Mol. Sci. 17:241. 10.3390/ijms17020241 26891297 PMC4783972

[B39] Fraguas D. Merchan-Náranjo J. Laita P. Parellada M. Moreno D. Ruiz-Sancho A.. (2008). Metabolic and hormonal side effects in children and adolescents treated with second-generation antipsychotics. J. Clin. Psychiatry 69, 1166–1175. 10.4088/JCP.v69n0717 18588363

[B40] Gabriel A. (2013). Risperidone, quetiapine, and olanzapine adjunctive treatments in major depression with psychotic features: a comparative study. Neuropsychiatr. Dis. Treat. 9, 485–492. 10.2147/NDT.S42745 23596349 PMC3627471

[B41] Gilula M. F. Barach P. R. (2004). Cranial electrotherapy stimulation: a safe neuromedical treatment for anxiety, depression, or insomnia. South Med. J. 97, 1269–1270. 10.1097/01.SMJ.0000136304.33212.06 15646771

[B42] Gunther M. Phillips K. D. (2010). Cranial electrotherapy stimulation for the treatment of depression. J. Psychosoc. Nurs. Ment. Health Serv. 48, 37–42. 10.3928/02793695-20100701-01 20669869

[B43] Hayes S. C. Hofmann S. G. (2017). The third wave of cognitive behavioral therapy and the rise of process-based care. World Psychiatry 16, 245–246. 10.1002/wps.20442 28941087 PMC5608815

[B44] Henry T. R. (2002). Therapeutic mechanisms of vagus nerve stimulation. Neurology 59, S3–S14. 10.1212/WNL.59.6_suppl_4.S3 12270962

[B45] Hincapie-Castillo J. M. Daniels P. F. (2017). Use of lithium in patients with unipolar depression. Lancet Psychiatry 4, 662–663. 10.1016/S2215-0366(17)30317-6 28853406

[B46] Holtzmann J. Richieri R. Saba G. Allaïli N. Bation R. Moliere F.. (2016). [How to define treatment-resistant depression?]. Presse Med. 45, 323–328. 10.1016/j.lpm.2016.02.002 26970938

[B47] Hunot V. Moore T. H. Caldwell D. M. Furukawa T. A. Davies P. Jones H. (2013). ‘Third wave’ cognitive and behavioural therapies versus other psychological therapies for depression. Cochrane Database Syst. Rev. CD008704 10.1002/14651858.CD008704.pub2 24142844 PMC12107518

[B48] Jiménez F. Nicolini H. Lozano A. M. Piedimonte F. Salín R. Velasco F. (2013). Electrical stimulation of the inferior thalamic peduncle in the treatment of major depression and obsessive compulsive disorders. World Neurosurg. 80, S30.e17– S30.e25. 10.1016/j.wneu.2012.07.010 22824558

[B49] Kato M. Chang C. M. (2013). Augmentation treatments with second-generation antipsychotics to antidepressants in treatment-resistant depression. CNS Drugs 27(Suppl. 1), S11–S19. 10.1007/s40263-012-0029-7 23709358

[B50] Kavirajan H. C. Lueck K. Chuang K. (2014). Alternating current cranial electrotherapy stimulation (CES) for depression. Cochrane Database Syst. Rev. CD010521. 10.1002/14651858.CD010521.pub2 25000907 PMC10554095

[B51] Kedzior K. K. Reitz S. K. Azorina V. Loo C. (2015). Durability of the antidepressant effect of the high-frequency repetitive transcranial magnetic stimulation (rTMS) In the absence of maintenance treatment in major depression: a systematic review and meta-analysis of 16 double-blind, randomized, sham-controlled trials. Depress. Anxiety 32, 193–203. 10.1002/da.22339 25683231

[B52] Kedzior K. K. Schuchinsky M. Gerkensmeier I. Loo C. (2017). Challenges in comparing the acute cognitive outcomes of high-frequency repetitive transcranial magnetic stimulation (HF-rTMS) vs. electroconvulsive therapy (ECT) in major depression: a systematic review. J. Psychiatr. Res. 91, 14–17. 10.1016/j.jpsychires.2017.03.002 28288306

[B53] Kellner C. (2014). Review: maintenance antidepressants reduce risk of relapse in the 6 months following ECT in people with major depression. Evid. Based Ment. Health 17:8. 10.1136/eb-2013-101663 24419098 PMC13404676

[B54] Kellner C. H. Greenberg R. M. Murrough J. W. Bryson E. O. Briggs M. C. Pasculli R. M. (2012). ECT in treatment-resistant depression. Am. J. Psychiatry 169, 1238–1244. 10.1176/appi.ajp.2012.12050648 23212054

[B55] Kellner C. H. Knapp R. G. Petrides G. Rummans T. A. Husain M. M. Rasmussen K.. (2006). Continuation electroconvulsive therapy vs. pharmacotherapy for relapse prevention in major depression: a multisite study from the Consortium for Research in Electroconvulsive Therapy (CORE). Arch. Gen. Psychiatry 63, 1337–1344. 10.1001/archpsyc.63.12.1337 17146008 PMC3708140

[B56] Kennedy S. H. Giacobbe P. Rizvi S. J. Placenza F. M. Nishikawa Y. Mayberg H. S.. (2011). Deep brain stimulation for treatment-resistant depression: follow-up after 3 to 6 years. Am. J. Psychiatry 168, 502–510. 10.1176/appi.ajp.2010.10081187 21285143

[B57] Kho K. H. van Vreeswijk M. F. Simpson S. Zwinderman A. H. (2003). A meta-analysis of electroconvulsive therapy efficacy in depression. J. ECT 19, 139–147. 10.1097/00124509-200309000-00005 12972983

[B58] Kirsch D. L. Nichols F. (2013). Cranial electrotherapy stimulation for treatment of anxiety, depression, and insomnia. Psychiatr. Clin. North Am. 36, 169–176. 10.1016/j.psc.2013.01.006 23538086

[B59] Kirsch D. L. Price L. R. Nichols F. Marksberry J. A. Platoni K. T. (2014). Military service member and veteran self reports of efficacy of cranial electrotherapy stimulation for anxiety, posttraumatic stress disorder, insomnia, and depression. US Army Med. Dep. J. 46–54. 25830798

[B60] Kito S. (2017). [Magnetic stimulation therapy for mood disorder]. Brain Nerve 69, 239–246. 10.11477/mf.1416200733 28270633

[B61] Kosel M. Schlaepfer T. E. (2003). Beyond the treatment of epilepsy: new applications of vagus nerve stimulation in psychiatry. CNS Spectr. 8, 515–521. 10.1017/S1092852900018988 12894032

[B62] Lasserre A. M. Strippoli M. F. Glaus J. Gholam-Rezaee M. Vandeleur C. L. Castelao E.. (2017). Prospective associations of depression subtypes with cardio-metabolic risk factors in the general population. Mol. Psychiatry 22, 1026–1034. 10.1038/mp.2016.178 27725658

[B63] Liebetanz D. Fregni F. Monte-Silva K. K. Oliveira M. B. Amâncio-dos-Santos A. Nitsche M. A.. (2006). After-effects of transcranial direct current stimulation (tDCS) on cortical spreading depression. Neurosci. Lett. 398, 85–90. 10.1016/j.neulet.2005.12.058 16448754

[B64] Lisanby S. H. Luber B. Schlaepfer T. E. Sackeim H. A. (2003). Safety and feasibility of magnetic seizure therapy (MST) in major depression: randomized within-subject comparison with electroconvulsive therapy. Neuropsychopharmacology 28, 1852–1865. 10.1038/sj.npp.1300229 12865903

[B65] Lozano A. M. Lipsman N. (2013). Probing and regulating dysfunctional circuits using deep brain stimulation. Neuron 77, 406–424. 10.1016/j.neuron.2013.01.020 23395370

[B66] Lucas N. Hubain P. Loas G. Jurysta F. (2017). Treatment resistant depression: actuality and perspectives in 2017. Rev. Med. Brux. 38, 16–25. 28525197

[B67] Manetti A. Hoertel N. Le Strat Y. Schuster J. P. Lemogne C. Limosin F. (2014). Comorbidity of late-life depression in the United States: a population-based study. Am. J. Geriatr. Psychiatry 22, 1292–1306. 10.1016/j.jagp.2013.05.001 23988281

[B68] Marangell L. B. Rush A. J. George M. S. Sackeim H. A. Johnson C. R. Husain M. M.. (2002). Vagus nerve stimulation (VNS) for major depressive episodes: one year outcomes. Biol. Psychiatry 51, 280–287. 10.1016/S0006-3223(01)01343-9 11958778

[B69] Maske U. E. Busch M. A. Jacobi F. Beesdo-Baum K. Seiffert I. Wittchen H. U.. (2015). Current major depressive syndrome measured with the Patient Health Questionnaire-9 (PHQ-9) and the Composite International Diagnostic Interview (CIDI): results from a cross-sectional population-based study of adults in Germany. BMC Psychiatry 15:77. 10.1186/s12888-015-0463-4 25884294 PMC4394554

[B70] McClintock S. M. Reti I. M. Carpenter L. L. McDonald W. M. Dubin M. Taylor S. F.. (2018). Consensus recommendations for the clinical application of repetitive transcranial magnetic stimulation (rTMS) in the treatment of depression. J. Clin. Psychiatry 79:16cs10905. 10.4088/JCP.16cs10905 28541649 PMC5846193

[B71] McCullough J. P. Jr. (2003). Treatment for chronic depression using Cognitive Behavioral Analysis System of Psychotherapy (CBASP). J. Clin. Psychol. 59, 833–846. 10.1002/jclp.10176 12858425

[B72] McLachlan R. S. (1997). Vagus nerve stimulation for intractable epilepsy: a review. J. Clin. Neurophysiol. 14, 358–368. 10.1097/00004691-199709000-00002 9415383

[B73] Meron D. Hedger N. Garner M. Baldwin D. S. (2015). Transcranial direct current stimulation (tDCS) in the treatment of depression: systematic review and meta-analysis of efficacy and tolerability. Neurosci. Biobehav. Rev. 57, 46–62. 10.1016/j.neubiorev.2015.07.012 26232699

[B74] Michaud C. M. Murray C. J. Bloom B. R. (2001). Burden of disease–implications for future research. JAMA 285, 535–539. 10.1001/jama.285.5.535 11176854

[B75] Minichino A. Bersani F. S. Capra E. Pannese R. Bonanno C. Salviati M.. (2012). ECT, rTMS, and deepTMS in pharmacoresistant drug-free patients with unipolar depression: a comparative review. Neuropsychiatr. Dis. Treat. 8, 55–64. 10.2147/NDT.S27025 22347797 PMC3280107

[B76] Mojtabai R. (2017). Nonremission and time to remission among remitters in major depressive disorder: revisiting STAR^*^D. Depress. Anxiety 34, 1123–1133. 10.1002/da.22677 28833903

[B77] Müller H. H. Kornhuber J. Maler J. M. Sperling W. (2013a). The effects of stimulation parameters on clinical outcomes in patients with vagus nerve stimulation implants with major depression. J. ECT 29, e40–e42. 10.1097/YCT.0b013e318290f7ed 23728236

[B78] Müller H. H. O. Lücke C. Moeller S. Philipsen A. Sperling W. (2017a). Efficacy and long-term tuning parameters of vagus nerve stimulation in long-term treated depressive patients. J. Clin. Neurosci. 44, 340–341. 10.1016/j.jocn.2017.06.020 28676315

[B79] Müller H. H. O. Reike M. Grosse-Holz S. Röther M. Lücke C. Philipsen A.. (2017b). Electroconvulsive therapy hasn't negative effects on short-term memory function, as assessed using a bedside hand-held device. Ment. Illn. 9:7093. 10.4081/mi.2017.7093 28748058 PMC5509960

[B80] Müller H. H. Reulbach U. Maler J. M. Kornhuber J. Sperling W. (2013b). Facilitative effects of VNS on the motor threshold: implications for its antidepressive mode of action? J. Neural. Transm. 120, 1507–1510. 10.1007/s00702-013-1043-8 23736944

[B81] Murphy J. A. Sarris J. Byrne G. J. (2017). A review of the conceptualisation and risk factors associated with treatment-resistant depression. Depress. Res. Treat. 2017:4176825. 10.1155/2017/4176825 28840042 PMC5559917

[B82] Nahas Z. Burns C. Foust M. J. Short B. Herbsman T. George M. S. (2006). Vagus nerve stimulation (VNS) for depression: what do we know now and what should be done next? Curr. Psychiatry Rep. 8, 445–451. 10.1007/s11920-006-0049-4 17094924

[B83] Nelson J. C. (2015). Adjunctive ziprasidone in major depression and the current status of adjunctive atypical antipsychotics. Am. J. Psychiatry 172, 1176–1178. 10.1176/appi.ajp.2015.15091220 26619770

[B84] Nelson J. C. Baumann P. Delucchi K. Joffe R. Katona C. (2014). A systematic review and meta-analysis of lithium augmentation of tricyclic and second generation antidepressants in major depression. J. Affect. Disord. 168, 269–275. 10.1016/j.jad.2014.05.053 25069082

[B85] Ng W. H. Donner E. Go C. Abou-Hamden A. Rutka J. T. (2010). Revision of vagal nerve stimulation (VNS) electrodes: review and report on use of ultra-sharp monopolar tip. Childs Nerv. Syst. 26, 1081–1084. 10.1007/s00381-010-1121-2 20225085

[B86] Nierenberg A. A. Alpert J. E. Gaynes B. N. Warden D. Wisniewski S. R. Biggs M. M.. (2008). Family history of completed suicide and characteristics of major depressive disorder: a STAR^*^D (sequenced treatment alternatives to relieve depression) study. J. Affect. Disord. 108, 129–134. 10.1016/j.jad.2007.10.011 18006073 PMC5886723

[B87] Oldham M. A. Ciraulo D. A. (2014). Bright light therapy for depression: a review of its effects on chronobiology and the autonomic nervous system. Chronobiol. Int. 31, 305–319. 10.3109/07420528.2013.833935 24397276 PMC5403163

[B88] Pagnin D. de Queiroz V. Pini S. Cassano G. B. (2004). Efficacy of ECT in depression: a meta-analytic review. J. ECT 20, 13–20. 10.1097/00124509-200403000-00004 15087991

[B89] Palm U. Ayache S. S. Padberg F. Lefaucheur J. P. (2016a). [Transcranial direct current stimulation (tDCS) for depression: results of nearly a decade of clinical research]. Encephale 42, 39–47. 10.1016/j.encep.2015.06.003 26216792

[B90] Palm U. Hasan A. Strube W. Padberg F. (2016b). tDCS for the treatment of depression: a comprehensive review. Eur. Arch. Psychiatry Clin. Neurosci. 266, 681–694. 10.1007/s00406-016-0674-9 26842422

[B91] Perera T. George M. S. Grammer G. Janicak P. G. Pascual-Leone A. Wirecki T. S. (2016). The Clinical TMS Society consensus review and treatment recommendations for TMS therapy for major depressive disorder. Brain Stimul. 9, 336–346. 10.1016/j.brs.2016.03.010 27090022 PMC5612370

[B92] Pinna M. Manchia M. Oppo R. Scano F. Pillai G. Loche A. P.. (2016). Clinical and biological predictors of response to electroconvulsive therapy (ECT): a review. Neurosci. Lett. 669, 32–42. 10.1016/j.neulet.2016.10.047 27793702

[B93] Post T. Kemmler G. Krassnig T. Brugger A. Hausmann A. (2015). [Efficacy of continuation and maintenance electroconvulsive therapy (c/m ECT) in the treatment of patients with therapy-resistant affective disorders: a retrospective analysis]. Neuropsychiatrie 29, 133–138. 10.1007/s40211-015-0150-1 26092747

[B94] Prudic J. (2008). Strategies to minimize cognitive side effects with ECT: aspects of ECT technique. J. ECT 24, 46–51. 10.1097/YCT.0b013e31815ef238 18379335

[B95] Rachid F. (2018). Maintenance repetitive transcranial magnetic stimulation (rTMS) for relapse prevention in with depression: a review. Psychiatry Res. 262, 363–372. 10.1016/j.psychres.2017.09.009 28951141

[B96] Rifkin A. (1988). ECT versus tricyclic antidepressants in depression: a review of the evidence. J. Clin. Psychiatry 49, 3–7. 3275634

[B97] Rizzo M. Creed F. Goldberg D. Meader N. Pilling S. (2011). A systematic review of non-pharmacological treatments for depression in people with chronic physical health problems. J. Psychosom. Res. 71, 18–27. 10.1016/j.jpsychores.2011.02.011 21665008

[B98] Rush A. J. Kraemer H. C. Sackeim H. A. Fava M. Trivedi M. H. Frank E.. (2006a). Report by the ACNP Task Force on response and remission in major depressive disorder. Neuropsychopharmacology 31, 1841–1853. 10.1038/sj.npp.1301131 16794566

[B99] Rush A. J. Trivedi M. H. Wisniewski S. R. Nierenberg A. A. Stewart J. W. Warden D.. (2006b). Acute and longer-term outcomes in depressed outpatients requiring one or several treatment steps: a STAR^*^D report. Am. J. Psychiatry 163, 1905–1917. 10.1176/ajp.2006.163.11.1905 17074942

[B100] Rutecki P. (1990). Anatomical, physiological, and theoretical basis for the antiepileptic effect of vagus nerve stimulation. Epilepsia 31(Suppl. 2), S1–S6. 10.1111/j.1528-1157.1990.tb05843.x 2226360

[B101] Sackeim H. A. Rush A. J. George M. S. Marangell L. B. Husain M. M. Nahas Z.. (2001). Vagus nerve stimulation (VNS) for treatment-resistant depression: efficacy, side effects, and predictors of outcome. Neuropsychopharmacology 25, 713–728. 10.1016/S0893-133X(01)00271-8 11682255

[B102] Sakurai H. Suzuki T. Yoshimura K. Mimura M. Uchida H. (2017). Predicting relapse with individual residual symptoms in major depressive disorder: a reanalysis of the STAR^*^D data. Psychopharmacology 234, 2453–2461. 10.1007/s00213-017-4634-5 28470399

[B103] Schlaepfer T. E. Lieb K. (2005). Deep brain stimulation for treatment of refractory depression. Lancet 366, 1420–1422. 10.1016/S0140-6736(05)67582-4 16243078

[B104] Schneider U. C. Bohlmann K. Vajkoczy P. Straub H. B. (2015). Implantation of a new Vagus Nerve Stimulation (VNS) Therapy(R) generator, AspireSR(R): considerations and recommendations during implantation and replacement surgery–comparison to a traditional system. Acta Neurochir. 157, 721–728. 10.1007/s00701-015-2362-3 25673257

[B105] Serafini G. Gonda X. Rihmer Z. Pompili M. Girardi P. Nasrallah H. A.. (2015). NMDA receptor antagonists for depression: critical considerations. Ann. Clin. Psychiatry 27, 213–220. 26247220

[B106] Serafini G. Howland R. H. Rovedi F. Girardi P. Amore M. (2014). The role of ketamine in treatment-resistant depression: a systematic review. Curr. Neuropharmacol. 12, 444–461. 10.2174/1570159X12666140619204251 25426012 PMC4243034

[B107] Shirzadi A. A. Ghaemi S. N. (2006). Side effects of atypical antipsychotics: extrapyramidal symptoms and the metabolic syndrome. Harv. Rev. Psychiatry 14, 152–164. 10.1080/10673220600748486 16787887

[B108] Sidhom E. Youssef N. A. (2014). Ultra-brief pulse unilateral ECT is associated with less cognitive side effects. Brain Stimul. 7, 768–769. 10.1016/j.brs.2014.06.013 25088459

[B109] Slepecky M. Kotianova A. Prasko J. Majercak I. Gyorgyova E. Kotian M.. (2017). Which psychological, psychophysiological, and anthropometric factors are connected with life events, depression, and quality of life in patients with cardiovascular disease. Neuropsychiatr. Dis. Treat. 13, 2093–2104. 10.2147/NDT.S141811 28831258 PMC5552144

[B110] Spielmans G. I. Berman M. I. Linardatos E. Rosenlicht N. Z. Perry A. Tsai A. C. (2013). Adjunctive atypical antipsychotic treatment for major depressive disorder: a meta-analysis of depression, quality of life, and safety outcomes. PLoS Med. 10:e1001403. 10.1371/journal.pmed.1001403 23554581 PMC3595214

[B111] Stage K. B. Kristoffersen J. Sørensen C. H. (2007). Lithium versus antidepressants in prevention of unipolar depression. A survey of a Cochrane review. Ugeskr Laeger 169, 3953–3955. 18078647

[B112] Sykes D. A. Moore H. Stott L. Holliday N. Javitch J. A. Lane J. R.. (2017). Extrapyramidal side effects of antipsychotics are linked to their association kinetics at dopamine D2 receptors. Nat. Commun. 8:763. 10.1038/s41467-017-00716-z 28970469 PMC5624946

[B113] Temmingh H. S. (2012). Extrapyramidal side-effects and antipsychotics: are second-generation agents still indicated? Br. J. Psychiatry 201:247. 10.1192/bjp.201.3.247 22945927

[B114] Tesio V. Marra S. Molinaro S. Torta R. Gaita F. Castelli L. (2017). Screening of depression in cardiology: a study on 617 cardiovascular patients. Int. J. Cardiol. 245, 49–51. 10.1016/j.ijcard.2017.07.065 28747268

[B115] The UK ECT Review Group (2003). Efficacy and safety of electroconvulsive therapy in depressive disorders: a systematic review and meta-analysis. Lancet 361, 799–808. 10.1016/S0140-6736(03)12705-5 12642045

[B116] Topfer L. A. Hailey D. (2001). Vagus nerve stimulation (VNS) for treatment-resistant depression. Issues Emerg. Health Technol. 25, 713–728. 11902224

[B117] Tschirdewahn J. Vignaud P. Pfeiffer A. Nolden J. Padberg F. Palm U. (2015). Transcranial direct current stimulation (tDCS) for the treatment of depression. MMW Fortschr. Med. 157, 46–48. 10.1007/s15006-015-7540-y 26977515

[B118] Uthman B. M. Wilder B. J. Hammond E. J. Reid S. A. (1990). Efficacy and safety of vagus nerve stimulation in patients with complex partial seizures. Epilepsia 31(Suppl. 2), S44–S50. 10.1111/j.1528-1157.1990.tb05849.x 2226366

[B119] Vandeleur C. L. Fassassi S. Castelao E. Glaus J. Strippoli M. F. Lasserre A. M.. (2017). Prevalence and correlates of DSM-5 major depressive and related disorders in the community. Psychiatry Res. 250, 50–58. 10.1016/j.psychres.2017.01.060 28142066

[B120] Warden D. Rush A. J. Wisniewski S. R. Lesser I. M. Kornstein S. G. Balasubramani G. K.. (2009). What predicts attrition in second step medication treatments for depression?: a STAR^*^D Report. Int. J. Neuropsychopharmacol. 12, 459–473. 10.1017/S1461145708009073 18611293 PMC5885751

[B121] Wiles N. Thomas L. Abel A. Barnes M. Carroll F. Ridgway N.. (2014). Clinical effectiveness and cost-effectiveness of cognitive behavioural therapy as an adjunct to pharmacotherapy for treatment-resistant depression in primary care: the CoBalT randomised controlled trial. Health Technol. Assess. 18, 1–167, vii–viii. 10.3310/hta18310 24824481 PMC4781198

[B122] Wright B. M. Eiland E. H. III. Lorenz R. (2013). Augmentation with atypical antipsychotics for depression: a review of evidence-based support from the medical literature. Pharmacotherapy 33, 344–359. 10.1002/phar.1204 23456734

[B123] Young A. H. (2013). Review: lithium reduces the risk of suicide compared with placebo in people with depression and bipolar disorder. Evid. Based Ment. Health 16, 112. 10.1136/eb-2013-101493 24046391

[B124] Zhou X. Ravindran A. V. Qin B. Del Giovane C. Li Q. Bauer M.. (2015). Comparative efficacy, acceptability, and tolerability of augmentation agents in treatment-resistant depression: systematic review and network meta-analysis. J. Clin. Psychiatry 76, e487–e498. 10.4088/JCP.14r09204 25919841

